# Staged Gamma Knife Radiosurgery for Large Skull Base Meningiomas

**DOI:** 10.7759/cureus.6001

**Published:** 2019-10-25

**Authors:** Yoshiyasu Iwai, Kazuhiro Yamanaka, Wataru Shimohonji, Kenichi Ishibashi

**Affiliations:** 1 Neurosurgery, Osaka City General Hospital, Osaka, JPN

**Keywords:** gamma knife, meningioma, radiosurgery, large, staged, skull base

## Abstract

Purpose: The authors have been treating skull base meningiomas using relatively low-dose gamma knife radiosurgery (GKS, ≤ 12 Gy) with acceptable tumor growth control and low morbidity. In the present study, volume-staged, low-dose GKS was performed for large skull base meningiomas with a maximum diameter > 4 cm. In this article, a treatment strategy for volume-staged GKS and results for large skull base meningiomas are described.

Methods: Data from 27 patients with large skull base meningiomas histopathologically diagnosed as WHO grade I or diagnosed by imaging, who underwent volume-staged GKS between March 1995 and September 2018, were reviewed. Among these patients, 24 were followed-up for > six months. The tumor was located in the parasellar region in nine patients, cavernous sinus region in four, petroclival region in four, petrocavernous sinus region in four, cerebellopontine angle region in two, and in the tent in one. The mean tumor diameters ranged from 31 to 47.8 mm (median 39.4 mm), with tumor volumes between 14.7 and 49.5 cm^3^ (median 27.5 cm^3^).

Results: The prescribed radiation dose was 8-12 Gy (median 10 Gy). The treatment interval between the first and second GKS was three to nine months (median 5.5 months). The median duration of follow-up after the first GKS was 84 months (range 6-204 months). Tumor volume decreased in nine (37.5%) patients, remained stable in nine (37.5%), and increased (local failure) in six (25%). The actuarial progression-free local control rate was 88% at three years, 78% at five years, 70% at 10 years, and 70% at 15 years. Neurological status improved in three (12.5%) patients, was unchanged in 16 (66.5%), and deteriorated in five (21%). Permanent radiation injury occurred in one (4%) patient.

Conclusion: Volume-staged GKS demonstrated the usefulness for large skull meningiomas > 4 cm in diameter, over a long-term follow-up period.

## Introduction

 In recent years, primary or adjuvant treatment using stereotactic radiosurgery (SRS) has gained favor for skull base meningiomas. SRS has demonstrated its safety and efficacy in the control of benign tumors, particularly for small to moderate size meningiomas. Tumor control rates for WHO grade I skull base meningiomas after SRS average approximately 91% and 88% at five and 10 years, respectively [1-5]. Most radiosurgical series for meningioma have excluded large-volume tumors. Traditionally, a tumor diameter of 30-35 mm has been the recommended cut-off for radiosurgery [6]. Recently, however, gamma knife radiosurgery (GKS) for large skull base meningiomas (> 8 cm^3^) has demonstrated an acceptable tumor growth control rate (84%), with worsening neurological function in 17% in mean follow-up of 6.5 years [7]. In that report, tumor volumes ≥14 cm^3^ still resulted in poor tumor control. We have been treating skull base meningiomas using relatively low-dose GKS (≤ 12 Gy), with acceptable tumor growth control and low morbidity [3]. As reported herein, we attempted to treat large skull base meningiomas with maximum diameters > 4 cm using low-dose volume-staged GKS. We discuss the utility and effectiveness of this method for the treatment of large skull base meningiomas in selected patients.

## Materials and methods

 Twenty-seven patients with large skull base meningiomas (maximum diameter > 4 cm) were treated using two-stage GKS (Elekta Instruments, Inc., Stockholm, Sweden) at the Osaka City General Hospital (Osaka, Japan) between March 1995 and September 2018. The Institutional Review Board of Osaka City General Hospital approved the research protocol. Among the 27 patients, 24 were followed-up for > six months and met the criteria for inclusion in this study and three patients were excluded due to below six months follow-up. There were nine males and 15 females, with an age range of 20-83 years (median 64.5 years). The tumor was located in the parasellar region in nine patients, cavernous sinus region in four, petroclival region in four, petrocavernous sinus region in four, cerebellopontine angle region in two, and in the tentorium based in one. Twelve (50%) patients were histologically diagnosed with WHO grade I meningiomas. The other 12 (50%) patients were diagnosed by MRI according to characteristic findings for meningiomas based on neuroimaging studies. Among the patients treated after surgical resection, seven (58%) were due to tumor regrowth and five (42%) were residual tumors. Among the patients without previous surgery, six (50%) were treated for tumor growth, and six (50%) were treated without tumor growth. Alteration in the function of cranial nerves II, III, IV, V, or VI was the most common neurological deficit on presentation. Mean tumor diameters ranged from 31 to 47.8 mm (median 39.4 mm), and mean tumor volumes were between 14.7 and 49.5 cm^3^ (median 27.5 cm^3^) (Table 1).

**Table 1:** Baseline characteristics of 24 patients with large skull base meningiomas treated with volume-staged GKS. GKS, gamma knife radiosurgery

Parameters	Values
Mean age (years, range)	64.5 (20-83)
Gender; male:female	9:15
Pre-GKS clinical manifestations	
Cranial nerve deficit	
Ⅱ	6(25%)
Ⅲ Ⅳ Ⅵ	8(33%)
Ⅴ	5(21%)
Ⅶ	1(4%)
Ⅷ	1(4%)
ⅨⅩ	0
Ⅺ	0
Ⅻ	0
Weakness	3(13%)
Ataxia	4(17%)
Cognitive impairment	3(13%)
Hypopituitarism	1(4%)
No symptoms	3(13%)
Location	
Parasellar	9(38%)
Cavernous sinus	4(16.5%)
Petroclival	4(16.5%)
Petrocavernous	4(16.5%)
Cerebellopontine angle	2(8.5%)
Tentorial based	1(4%)
Tumor volume	
Median (range; ㎤)	27.5(14.7-49.5)
Mean tumor diameter	
Median (range; mm)	39.4(31-47.8)
Median follow-up duration (range, months)	84(6-204)

 Indications for two-stage radiosurgery were as follows. Tumor size in all patients was > 40 mm in maximum diameter. GKS was performed in two sessions separated by a six-month interval. The second GKS was performed using references from the first GKS dose plan. Dose planning was performed on the Gamma plan and, for the second treatment, referred to the previous computer dose plan, which minimized the overlap of radiation. Radiosurgical treatment was basically designed to divide the tumor into upper and lower portions. The reason for this divided plan was that the dose distribution of the gamma knife is elliptical in shape, and the Z axis is shorter than the X and Y axes [8]. Therefore, if the radiation dose is divided into the upper and lower parts of the tumor, the overlap of radiation to the surrounding neural structures can be minimized. The determination of which part to treat first depends on tumor location and extension. Basically, the authors planned to treat parts of the tumor that were closer to critical organs such as the optic apparatus or brain stem. Patients were routinely followed clinically and radiologically every six months for three years, and then annually after GKS. At each follow-up visit, a neurological examination was performed to evaluate for new neurological deficits and for neuroimaging studies. Tumor size was defined as any decrease in maximum diameter and any increase in maximum diameter. The presence of peritumoral edema was defined as a newly appearing high signal on T2-weighted imaging. Clinically, the improvement or deterioration of pre-existing symptoms, and the presence of newly appearing neurological symptoms, were investigated. Data are expressed as median or mean and range for continuous variables, and as frequency and percentage for categorical variables. Statistical analyses of categorical variables were performed using the chi-squared and Fisher’s exact tests. The tumor-free progression was assessed in univariate analysis using log-rank test. Differences with p < 0.05 were considered to be statistically significant.

## Results

**Figure 1:**
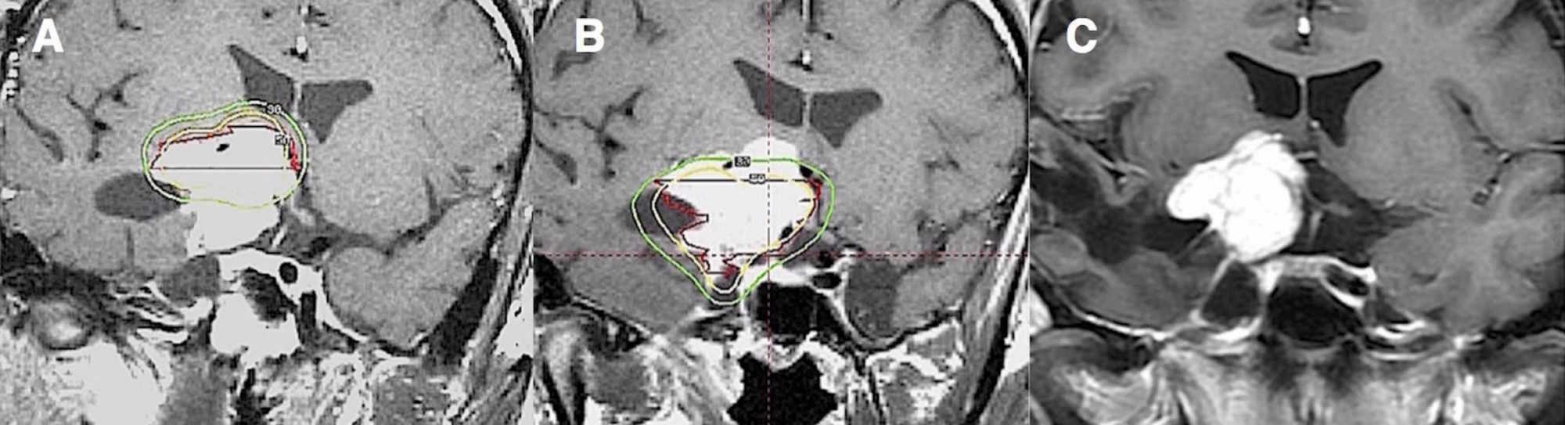
Coronal MRI revealing a sphenoidal meningioma in a 19-year-old male after surgical resection. A: Treatment dose planning MRI for the first gamma knife radiosurgery reporting a tumor marginal dose of 10 Gy (50% isodose) to the upper part of the tumor.  B: Treatment dose planning MRI for the second gamma knife radiosurgery reporting a tumor marginal dose of 10 Gy (50% isodose) to the lower part of the tumor.  C. MRI data acquired 13 years after gamma knife radiosurgery demonstrating a decrease in tumor size.

The prescribed marginal dose was 8-12 Gy (median 10 Gy). The prescribed marginal dose was 8 Gy in 10 patients, 10 Gy in 13, and 12 Gy in one. The prescribed isodose line was 50% in all patients. The treatment interval between the first and second GKS was three to nine months (median 5.5 months). The upper part of the tumor was treated first in 19 (79%) patients and the lower part was first treated in five (21%) (Figure 1). 

The treatment volume was 6.3-26.4 cm^3^ (median 12.7 cm^3^) (Table 2). 

**Table 2:** Parameters of volume-staged GKS. GKS, gamma knife radiosurgery

Parameters	Values
Median treatment volume, range (㎤)	12.7, 6.3-26.4
Median marginal dose, range (Gy)	10, 8-12
Median isodose line (%)	50
Interval of staged GKS	
Median, range (months)	5.5, 3-9

**Figure 2:**
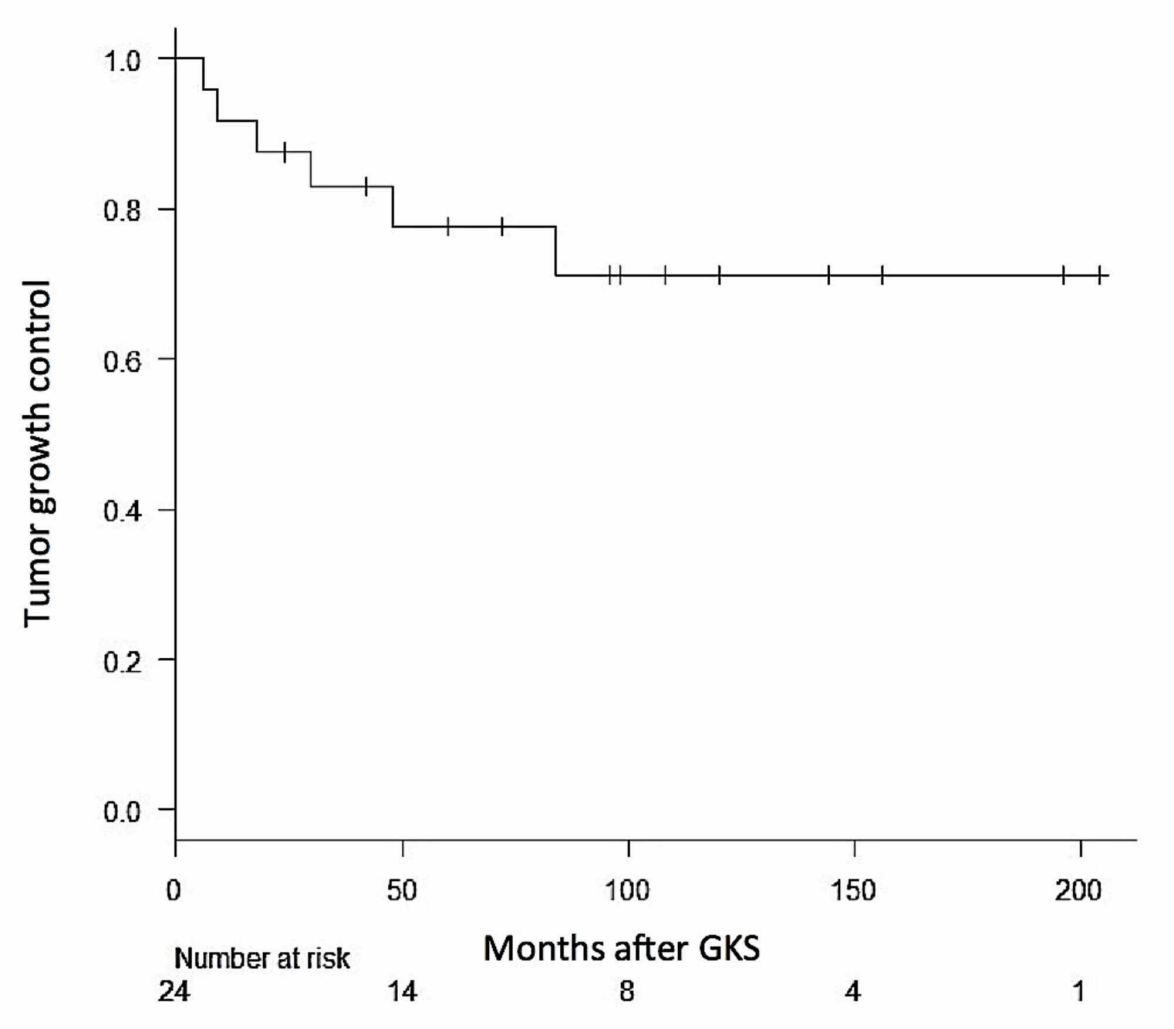
Kaplan-Meier curve for local tumor growth control in 24 patients treated with staged GKS for large skull base meningioma. GKS, gamma knife radiosurgery

The mean duration of follow-up after the first GKS was 84 months (range 6-204 months). Tumor volume decreased in nine (37.5%) patients, remained stable in nine (37.5%), and increased (local failure) in six (25%). Local tumor control was achieved in 16 (75%) patients. The actuarial progression-free local control rate was 88% at three years, 78% at five years, 70% at 10 years, and 70% at 15 years (Figure 2). 

One patient experienced out-of-field recurrence in the skull base region, and overall tumor growth control was 88% at three years, 76% at five years, 70% at 10 years, and 70% at 15 years. Using the log-rank test of independence, patient age, sex, previous surgery, radiation dose to the tumor margin, and tumor volume were not statistically significant factors in predicting local tumor control (Table 3).

**Table 3:** Predictors of local tumor growth control.

Variable	Univariate
	p value
Age ≧ 60 y	0.3897
Male gender	0.7971
PreGKS op(-)	0.7037
Tumor volume <25 ㎤	0.6154
Marginal dose ≧ 10Gy	0.5459

 Neurological status improved in three (12.5%) patients, was unchanged in 16 (66.5%), and deteriorated in five (21%). Among patients who experienced clinical improvement, one with sphenoid ridge meningioma exhibited improved visual disturbance, one with petroclival meningioma experienced improved cerebellar ataxia, and one with sphenoid cavernous meningioma experienced improvement in disorientation. Among signs of neurological deterioration, three (13%) patients exhibited tumor progression and two (8%) experienced radiation injury. Among the radiation injuries, one patient with large petrocavernous sinus meningioma exhibited mild deterioration of pre-existing oculomotor nerve palsy 60 months after GKS. One patient with a large sphenoidal meningioma and perifocal edema before GKS exhibited deteriorated perifocal edema and disturbance in consciousness. Three patients died due to tumor growth and one from unrelated renal cell carcinoma. Radiation injury to the surrounding brain tissue occurred in two (8%) patients, of whom one with petroclival meningioma experienced transient deterioration of cerebellar ataxia 24-36 months after GKS. One patient with large sphenoid wing meningioma with perifocal edema required surgical resection 11 months after GKS due to progression of perifocal edema and deterioration of consciousness. One patient with sphenocavernous sinus meningioma required surgery 40 months after GKS and exhibited malignant transformation histologically. One patient required ventriculo-peritoneal shunt due to hydrocephalus three months after GKS due to pre-existing hydrocephalus. One patient with shenocavernous sinus meningioma required GKS for orbital extension 13 years after the first GKS and skull base extension at 20 years after the first GKS. One patient with petroclival meningioma underwent surgery 84 months after GKS due to tumor growth.

## Discussion

Published studies investigating the long-term efficacy of radiosurgery for skull base meningioma have reported tumor control rates of 89%-98%, and acceptable complication rates of 4%-14% [9-13]. Our group previously reported the long-term outcome of single-session GKS for skull base meningiomas [3]. The five-year actuarial tumor control rate was 93%, and unfavorable outcome of cranial neuropathy occurred in 6%. Improvement in cranial neuropathy was 15%, even with a low marginal dose of 12 Gy. Stratifying meningiomas according to size is expected to yield different GKS planning and prognostic outcomes, especially in confined areas such as the skull base. Investigators at The University of Pittsburgh (Pittsburgh, Pennsylvania) found that tumor volume > 8 cm^3^ was the most important factor associated with poorer prognosis for radiosurgery of benign meningiomas [14-17]. The results for single-session radiosurgery for large skull base meningiomas (> 8 cm^3^) reported five-year actuarial tumor control in 88.6%; however, ≥ 14 cm^3^ tumor volume is worse tumor control than < 14 cm^3^ tumor volume (five-year tumor control, 80% vs. 100%; 10-year tumor control, 73% vs. 95%) [7]. In the present study, even with a median tumor diameter of 27.5 cm^3^ (range, 14.7-49.5 cm^3^) and using a median prescribed dose of 10 Gy (range, 8-10 Gy), we could achieve five-year tumor growth control in 78%, 10 years in 70%, with only 6% experiencing permanent radiation injury with 84 months of follow-up. Another option for radiation therapy for large skull base meningiomas is fractionated stereotactic radiotherapy (FSRT). Five-year tumor growth control rates were reported to be 93%-96%, with clinical late toxicity of 1.6%-5.5%, using 50-56.8 Gy for tumor volumes of 35.4-52.5 cm^3^, with mean follow-up of 35-42 months [18-19]. A recent study compared single-session GKS (n = 42) with fractionated GKS (FGKS) (n = 28) for meningiomas with tumor volumes > 10 cm^3^ [20]. The FGKS group also included 16 skull base meningiomas. The mean tumor volume for single-session GKS and FGKS was 15.2 and 21 cm^3^, respectively. The FGKS group demonstrated a higher overall five-year tumor control rate than the single-session GKS group (92.9% vs. 88.1%, respectively); the difference, however, was not statistically significant (p = 0.389). Recently, Su et al. described a volume-staged GKS technique similar to ours called the “snowman-shape” design [21]. They adopted this technique for four patients with large skull base meningiomas. They treated the large basal portion with the prescribed dose of 13.5 Gy (12-15 Gy), and the upper part of the tumor, close to the optic apparatus, using 9 Gy (8-10 Gy). They achieved 100% tumor growth control without adverse radiation injury with 100.5 months’ follow-up. The difference between their strategy and ours was that they treated the lower portion of the tumor first, while we treated the tumor close to critical structures, mainly the upper part of the tumor. Although we do not disagree with their strategy of first irradiating the basal tumor close to the tumor attachment with vascular supply, we consider the risk for clinical deterioration during the wait for the second GKS treatment due to tumor growth. As such, we first treat the tumor close to critical structures, which is often the upper part of tumors. We consider our volume-staged GKS protocol to be effective, especially given the sharp decline in radiation dose, which has significant advantages for benign skull base meningiomas requiring long-term follow-up (up to 10 years) to consider delayed radiation injury to surrounding critical structures. The present investigation was a retrospective study and we did not compare the results of FSRT in a randomized analysis, or patients involved from the beginning of this strategy in the 1990s. Although innovations in gamma knife technology and dose plan computing were not addressed in this study, we believe that differences in treatment outcomes between treatment periods are not significant.

## Conclusions

 Volume-staged GKS using low-dose radiosurgery demonstrated the usefulness for large skull meningiomas, even those > 4 cm in diameter, over a long-term follow-up period. We continue effort to improve the technique because there is certainly a space to get more control with GKS in these voluminous tumors.
